# Is the proportional recovery rule applicable to the lower limb after a first-ever ischemic stroke?

**DOI:** 10.1371/journal.pone.0189279

**Published:** 2018-01-12

**Authors:** Janne M. Veerbeek, Caroline Winters, Erwin E. H. van Wegen, Gert Kwakkel

**Affiliations:** 1 Department of Rehabilitation Medicine, VU University Medical Center, Amsterdam, The Netherlands; 2 Amsterdam Movement Sciences, Amsterdam, the Netherlands; 3 Amsterdam Neuroscience, Amsterdam, the Netherlands; 4 Division of Vascular Neurology and Neurorehabilitation, University of Zurich, Zurich, Switzerland; 5 Cereneo, Center for Neurology and Rehabilitation, Vitznau, Switzerland; 6 Rehabilitation Research Center, Reade, Amsterdam, the Netherlands; 7 Department of Physical Therapy and Human Movement Sciences, Northwestern University, Chicago, Illinois, United States of America; UniversitatsKlinikum Heidelberg, GERMANY

## Abstract

**Objective:**

To investigate (a) the applicability of the proportional recovery rule of spontaneous neurobiological recovery to motor function of the paretic lower extremity (LE); and (b) the presence of fitters and non-fitters of this prognostic rule poststroke. When present, the clinical threshold for fitting nor non-fitting would be determined, as well as within-subject generalizability to the paretic upper extremity (UE).

**Methods:**

Prospective cohort study in which the Fugl-Meyer Assessment (FMA)-LE and FMA-UE were measured <72 hours and 6 months poststroke. Predicted maximum potential recovery was defined as [FMA-LE_max_−FMA-LE_initial_ = 34 –FMA-LE_initial_]. Hierarchical clustering in 202 first-ever ischemic stroke patients distinguished between fitting and not fitting the rule. Descriptive statistics determined whether fitters and non-fitters for LE were the same persons as for UE.

**Results:**

175 (87%) patients fitted the FMA-LE recovery rule. The observed average improvement of the fitters was ~64% of the predicted maximum potential recovery. In the non-fitter group, the maximum initial FMA-LE score was 13 points. Fifty-one out of 78 patients (~65%) who scored below the identified 14-point threshold at baseline fitted the FMA-LE rule. Non-fitters were more severely affected than fitters. All non-fitters of the FMA-LE rule did also not fit the proportional recovery rule for FMA-UE.

**Conclusions:**

Proportional recovery seems to be consistent within subjects across LE and UE motor impairment at the hemiplegic side in first-ever ischemic hemispheric stroke subjects. Future studies should investigate prospectively distinguishing between fitters and not-fitters within the subgroup of patients who have initial low FMA-LE scores. Subsequently, patients could be stratified based on fitting or not fitting the recovery rule as this would impact rehabilitation management and trial design.

## Introduction

It is suggested that about 90% of all neurological improvement during the first 6 months after stroke is defined by progress of time alone [1]. However, the neurophysiological mechanisms driving neurobiological recovery are poorly understood [2]. Prospective studies showed that the amount of neurobiological recovery of the paretic upper extremity (UE) [3–6], visuo-spatial neglect (VSN) [7], and speech [8] is relatively fixed–ranging from 60 to 97%–and highly predictable. Proportional recovery is the percentage that a patient improves over time for a specific measure, such as the Fugl-Meyer Assessment (FMA) motor score, in relation to his or her available maximum improvement on that specific measure. Patients with first-ever *right* hemispheric lesions who do not follow the proportional recovery rule (i.e., ‘non-fitters’, patients who improve to a lesser extent on a specific measure than would have been expected based on the proportional recovery rule) for one modality such as motor recovery of the upper extremity are also likely not to follow the rule on other modalities such as VSN [7]. This suggests that mechanisms driving spontaneous neurobiological recovery poststroke generalize across neurological impairments. Recently, in a small cohort of 32 patients, it was shown that the proportional recovery rule is also applicable to motor function of the paretic lower extremity (LE) [9]. However, the lack of non-fitters following the proportional recovery rule in this specific cohort was an unsuspected finding. The authors concluded that the absence of a non-fitter group may be caused by differences in the neuroanatomical organization of descending motor tracts to the lower limb when compared to the upper limb [9].

The present study therefore aimed to investigate the generalizability of the ‘proportional recovery rule’ for motor function of the paretic upper extremity, measured with the FMA-UE subscale, to motor function of the paretic lower extremity, measured with the FMA-LE subscale within 72 hours and at 6 months poststroke in a considerably larger cohort of first-ever ischemic hemispheric stroke patients. This included investigating the presence of both fitters and non-fitters of the proportional recovery rule. When present, the secondary aims were to determine whether (a) there was a clinical threshold for the FMA-LE within 72 hours, separating non-fitters from fitters; (b) fitters and non-fitters could be distinguished based on demographic and clinical characteristics at baseline; and (c) fitters or non-fitters of the proportional recovery rule for the lower extremity were the same patients who do or do not show proportional recovery for the paretic upper extremity.

## Materials and methods

Data from the prospective cohort of the Early Prediction of functional Outcome after Stroke (EPOS) study were used. Details of this study have been published elsewhere [5, 10]. Stroke patients were included when they met the following criteria: (1) first-ever ischemic anterior circulation stroke in one hemisphere; (2) mono- or hemiparesis within 72 hours after onset; (3) premorbid Barthel Index score 19 or more out of 20; (4) aged 18 years or older; (5) no severe deficits of communication, memory, or understanding; and (6) written informed consent. For this study, only patients were included who had a FMA-LE motor score of less than 34 (i.e., a lower limb paresis), a FMA-UE motor score of less than 66 (i.e., an upper limb paresis) within 72 hours poststroke, and with available FMA-LE and UE scores at 6 months poststroke.

Ethical approval was obtained before start of participant recruitment from the nationally certified Ethical Committee of the VU University Medical Center, Amsterdam, the Netherlands (https://www.vumc.nl/afdelingen/METc/METc/). Local feasibility was approved by the institutional review boards of the participating hospitals (AMC, Amsterdam; Erasmus MC, Rotterdam; LUMC, Leiden; UMC Sint Radboud, Nijmegen; UMC Utrecht; Amphia Hospital Breda; Diaconessen Hospital, Leiden; Franciscus Hospital, Roosendaal) and nursing homes (Sint Jacob, Amsterdam; Zonnehuis, Amsterdam; Cordaan/Berkenstede, Amsterdam; Laurens Antonius Binnenweg, Rotterdam; Reumaverpleeghuis, Rotterdam; Albert van Koningsbruggen, Utrecht; Wiekendaal, Roosendaal). The capacity to consent was determined during the screening and consent visits. This was based on the patients’ ability to (1) understand the participant information (oral and written); (2) explain why they were admitted to the hospital; and (3) follow two-staged commands as requested in the Mini-Mental State Examination.

The FMA-LE (score range 0–34) and FMA-UE (score range 0–66) subscales were measured within 72 hours and at 6 months after stroke onset. The FMA quantifies limb impairment in terms of synergistic (in)dependent motor control [11]. Observed motor recovery of the lower extremity was defined as [ΔFMA-LE_observed_ = FMA-LE_6 months_−FMA-LE_initial_] and predicted maximum potential recovery as [FMA-LE_max_−FMA-LE_initial_ = 34 –FMA-LE_initial_].

Hierarchical clustering analysis based on the average pairwise Mahalanobis distances method was used to classify patients into fitters and non-fitters of the proportional recovery rule (Matlab’s Statistic toolbox, version 8.1, Matlab version 2013a, Mathwords Inc, Natwick, MA). We selected the Mahalanobis distance method and not the more common used Euclidian distance with circular boundaries, as it also takes co-variances into account and leads to elliptic decision boundaries. Fitters were defined as patients who showed a comparable amount of predicted maximum potential and observed improvement on the FMA-LE. Patients who did not show this comparable amount of predicted maximum potential and observed improvement were considered non-fitters. Goodness-of-fit was assessed by the cophenetic correlation and the Spearman correlation between the Mahalanobis and cophenetic distances obtained from the dendogram. Linear regression was applied to determine the percentage of the predicted maximum potential recovery (R^2^) that explained the observed change of the lower extremity in the fitter subgroup. Normality of data was checked by visual inspection of histograms. Patient characteristics were analyzed by descriptive statistics. Differences between fitters and non-fitters by the independent *t* test for parametric data, Pearson’s X^2^ test for categorical data, and the Mann-Whitney U test for nonparametric data.

To assess the threshold value on the initial FMA-LE for not fitting the proportional recovery rule, the highest score of the initial FMA-LE in the non-fitter subgroup was taken. The sensitivity, specificity, positive predictive value, and negative predictive value of this threshold were determined. In a next step, characteristics were compared between patients who scored below this threshold value but still fitted the rule and those who did not. SPSS (version 22) was used unless indicated otherwise and a two-tailed P-value <0.05 was considered statistically significant.

Predicted maximum potential recovery and observed change for the upper extremity were defined as [FMA-UE_max_−FMA-UE_initial_ = 66 –FMA-UE_initial_] and [ΔFMA-UE_observed_ = FMA-UE_6 months_−FMA-UE_initial_] [3]. Subsequently, ΔFMA-LE_observed_ and ΔFMA-UE_observed_ were expressed as percentages of their maximum possible scores in order to compare the distribution of maximum potential and observed recovery. Descriptive statistics were used to determine whether fitters and non-fitters were the same persons for both outcomes.

## Results

**Fig 1:**
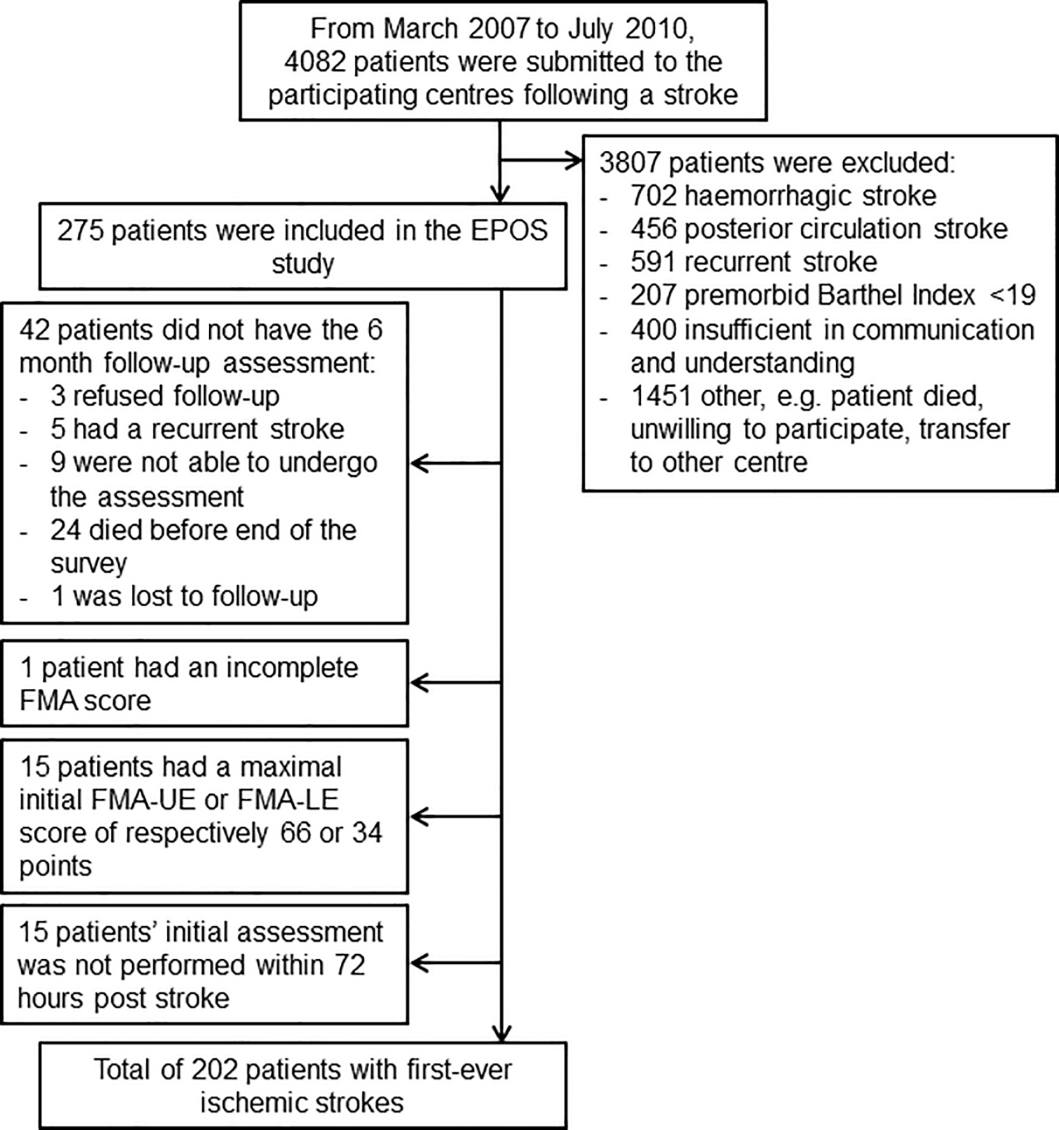
Exclusion flow-chart.

**Fig 2:**
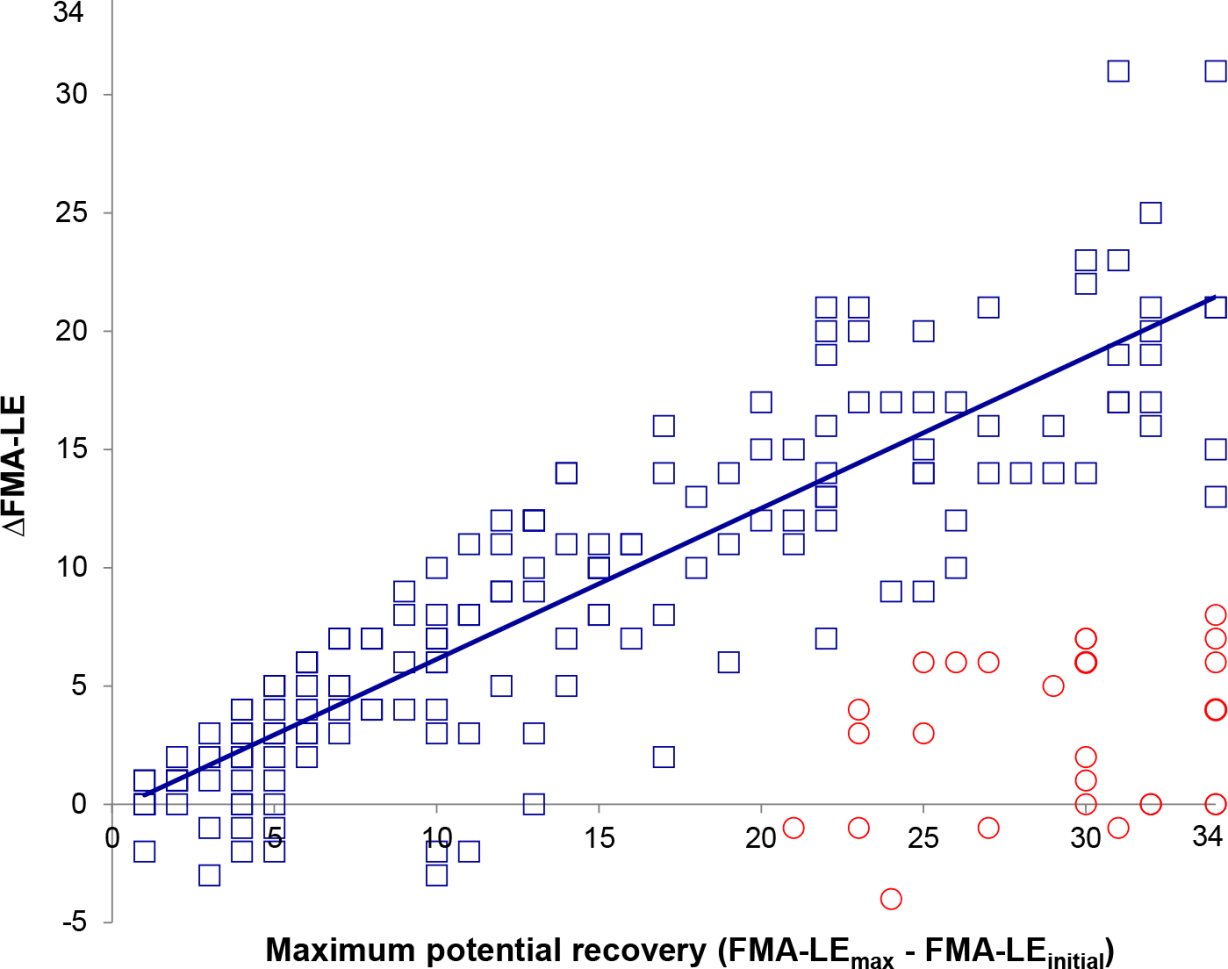
Proportional recovery of the lower extremity: Predicted maximum potential recovery (FMA-LE_max_−FMA-LE_initial_) versus observed ΔFMA-LE. ◻ subgroup of fitters (N = 175; blue), ○ subgroup of non-fitters (N = 27; red). For the fitters, R^2^ of the FMA-LE_max_ for ΔFMA-LE was 76.8%, and the regression line y = 0.64 (95%CI 0.59–0.69) x– 0.24 (95%CI -1.15–0.66). Note that the two data points at the top right corner were also identified as a subgroup in the hierarchical cluster analysis. However, they were added to the ‘fitter’ group because their ΔFMA-LE_observed_ was almost identical with the predicted maximum potential recovery. Also, note that there were 15 patients who scored 1 to 3 points lower on the FMA-LE at 6 months, in comparison to the baseline measurement. See further explanation in text. CI, Confidence Interval; FMA, Fugl-Meyer Assessment; LE, Lower Extremity.

A total of 202 patients met the present inclusion criteria (Fig 1). The mean age of the total sample was 66.62 (standard deviation [SD] 13.97) years, 106 (52.5%) subjects were male, and the mean National Institutes of Health Stroke Scale (NIHSS) score within 72 hours was 9.27 (SD 5.78) points. Hierarchical clustering analysis showed that 175 patients (86.6%) fitted the FMA-LE proportional recovery rule (Fig 2). The goodness-of-fit was c = 0.73 (i.e., cophenetic correlation coefficient) and r_s_ = 0.80 (Spearman correlation). Two patients had high predicted maximum potential recovery and observed recovery (data points at the top right corner of Fig 2). These patients were also characterized as ‘outliers’ in the hierarchical cluster analysis. However, as their predicted and observed recovery matched, they were added to the ‘fitters’ group. Note that there were patients who had lower scores at follow-up, in comparison to their initial FMA-LE score, which resulted in a negative ΔFMA-LE_observed_ (see Fig 2). As these patients were part of the groups based on the hierarchical clustering analysis and the decline in FMA-LE score was within the measurement error of the FMA-UE, we did not exclude these patients from analyses.

For the fitters, the median FMA-LE maximum potential recovery was 12 (interquartile range [IQR] 6–22) points and ΔFMA-LE_observed_ was 8 (IQR 3–14). For the non-fitters, these were 30 (IQR 25–32) and 3 (IQR 0–6), respectively. The observed improvement of the fitters was ~64% (95% confidence interval [CI] 59–69%) of the predicted maximum potential recovery (i.e., proportional recovery). At baseline, fitters had significantly lower neurological impairments and less motor impairment when compared to non-fitters (p < .001; Table 1). In addition, predicted maximum potential recovery of both FMA-LE and FMA-UE was significantly higher in fitters (P < .001).

**Table 1:** Group comparison regarding patient characteristics (N = 202).

Determinant (<72 hours poststroke)	Fitters (N = 175)	Non-fitters (N = 27)	P
Age, years*	65.71 (14.15)	72.56 (11.15)	.160
Gender, male/female	85/90	11/16	.448
Hemisphere of stroke, right/left	97/78	18/7	.372
Recombinant tissue plasminogen activator, yes/no	42/133	11/16	.066
Time between stroke onset and			
initial assessment, days*	2.06 (0.81)	1.85 (1.03)	.061
6-month assessment, days*	188.87 (12.75)	184.59 (17.70)	.603
Bamford classification, LACI/PACI/TACI	91/53/31	1/13/13	< .001
CIRS initial†	2 (1–4)	2 (1–3)	.769
Cardiac disorders, yes/no	55/120	11/16	.337
Vascular disorders, yes/no	51/124	9/18	.657
Endocrine and metabolic disorders, yes/no	43/132	4/23	.264
NIHSS initial†	7 (4–12)	17 (15–20)	< .001
Hemianopia, yes/no	43/132	20/7	< .001
Sensory loss, yes/no	101/74	26/1	< .001
Inattention, yes/no	68/107	21/6	< .001
FMA-LE			
<72 hours†	22 (12–28)	4 (2–9)	< .001
6 months†	30 (26–33)	8 (4–12)	< .001
Maximum potential recovery†	12 (6–22)	30 (25–32)	< .001
ΔFMA-LE_observed_†	8 (3–14)	3 (0–6)	< .001
FMA-UE			
<72 hours†	23 (7–52)	4 (2–5)	< .001
6 months†	60 (48–65)	7 (4–9)	< .001
Maximum potential recovery†	43 (14–59)	62 (61–64)	< .001
ΔFMA-UE_observed_†	17 (7–37)	2 (0–5)	< .001

Abbreviations: CIRS, Cumulative Illness Rating Scale; FMA, Fugl-Meyer Assessment; LACI, Lacunar Anterior Circulation Infarcts; LE, Lower Extremity; NIHSS, National Institutes of Health Stroke Scale; PACI, Partial Anterior Circulation Infarcts; TACI, Total Anterior Circulation Infarcts; UE, Upper Extremity; Δ, change.

*, mean (standard deviation)

†, median (interquartile range)

The maximum initial FMA-LE score within the non-fitter group (N = 27) was 13 points (38% of the total score). Overall, 78 patients had a FMA-LE score of 13 points or lower at baseline. In this subgroup, 51 (~65%) patients fitted the rule for the lower extremity and 27 (~35%) did not. The sensitivity was 0.71 (95%CI 0.63–0.77), the specificity 1.00 (95%CI 0.84–1.00), the positive predictive value 1.00 (95%CI 0.96–1.00), and the negative predictive value 0.34 (95%CI 0.24–0.46). The non-fitters were more severely affected than the fitters, as indicated by the initial NIHSS (17 [IQR 15–20] and 13 [IQR 9–17], respectively; P = .001) and Bamford classification (lacunar anterior circulation infarcts [LACI] = 1, partial anterior circulation infarcts [PACI] = 13, total anterior circulation infarcts [TACI] = 13 vs. LACI = 20, PACI = 15, TACI = 16, respectively; P = .008).

**Fig 3:**
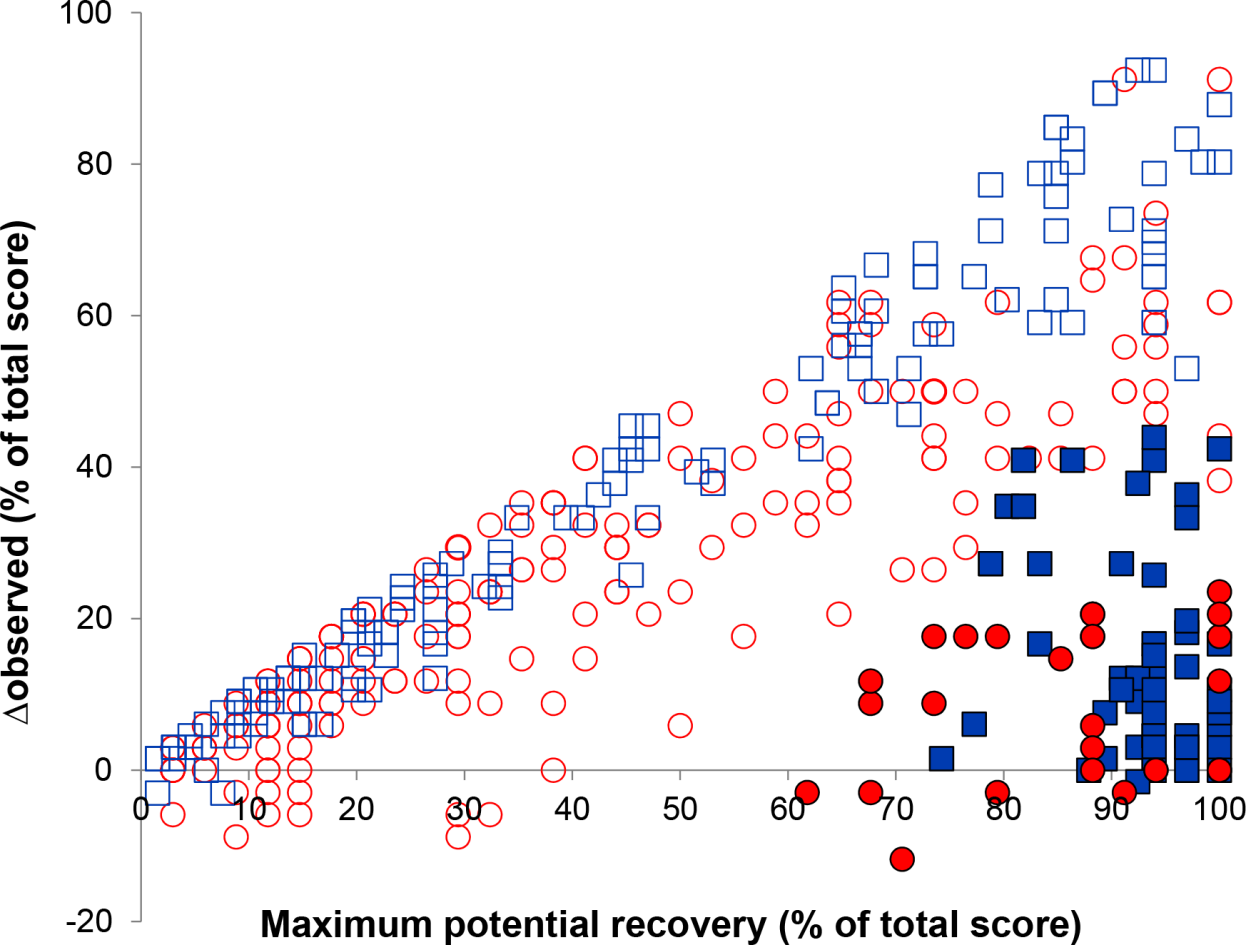
Consistency of proportional recovery between FMA-LE and FMA-UE expressed in percentages (%). ◻ subgroup of FMA-UE fitters (N = 137; blue), ◼ subgroup of FMA-UE non-fitters (N = 65; blue), ○ subgroup of FMA-LE fitters (N = 175; red), ● subgroup of FMA-LE non-fitters (N = 27; red). FMA, Fugl-Meyer Assessment; LE, Lower Extremity; UE, Upper Extremity.

Comparing fitters and non-fitters for both the predicted maximum potential and observed ΔFMA-LE showed the same pattern as for the maximum potential and observed ΔFMA-UE (Fig 3). All non-fitters of FMA-LE (N = 27) also did not fit the rule for FMA-UE. Thirty-eight (21.7%) of the FMA-LE fitters did not fit the rule for FMA-UE. Conversely, none of the patients that were non-fitters on FMA-UE fitted the rule for FMA-LE.

## Discussion

The present prospective cohort study confirmed that the proportional recovery rule is generalizable to motor function of the paretic lower extremity in patients with a first-ever ischemic hemispheric stroke. Patients who fitted this rule improved on average 64% (95% CI 59–69%) of their predicted maximum potential recovery. In addition, the present study shows that also for motor function of the lower extremity, there seems to be a subgroup of patients who did not fit the proportional recovery rule (i.e., non-fitters). These non-fitters are characterized by having more neurological impairments such as hemianopia, VSN, and sensory loss, when compared to patients who did follow the rule (i.e., fitters). Importantly, *all* patients who had an initial FMA-LE score of 14 points or higher within 72 hours poststroke did follow the proportional recovery rule, whereas only 35% of the patients with scores below this critical threshold failed to follow the expected amount of spontaneous neurobiological recovery. This finding also suggests that even stroke patients with a very severe lower extremity deficit (i.e., below 14 points on the FMA-LE) at stroke onset may show a tremendous amount of spontaneous neurobiological improvement of up to 20 out of 34 points on the FMA-LE. The proportion of non-fitters was about 13% (N = 27) of our total cohort. This critical threshold of 14 points is in line with the threshold found for non-fitters of the FMA-UE (i.e., less than 17 points) [5] and suggests that there are common threshold-dependent mechanisms which define proportional recovery within the first days after a first-ever ischemic stroke. Moreover, hemiplegic patients who were non-fitters of motor function of the paretic lower extremity (N = 27) were also non-fitters on the proportional recovery rule of the paretic upper extremity.

Our findings are in line with previous studies about the proportional recovery rule of motor function of the upper [3–6] and lower extremity [9], speech [8], and VSN [7]. However, in contrast to the recent published study of Smith and co-workers [9], the present larger cohort did include also more severely affected hemiplegic stroke patients with an unfavorable prognosis for recovery of gait [10]. Obviously, a number of these patients with a poor prognosis for gait did not follow the proportional recovery rule (i.e., non-fitters) of spontaneous neurobiological recovery after stroke. With that, we suggest that spontaneous neurobiological recovery is a consistent intra-hemispheric phenomenon that seems to occur irrespective of the type of neurological impairments poststroke. In addition, this ‘70% recovery rule’ is not fixed, but may show variance ranging from 64% for motor recovery of the paretic lower extremity (95%CI 59–69%) in the present study to 97% (95%CI 82–112) for VSN [7]. At least, the result from the current and previous cohort studies in this field further confirm that proportional recovery is inherent to acute stroke and reflects common underlying mechanisms of spontaneous neurobiological recovery. The hypothesis of no direct corticospinal involvement in the lower extremity by the assumed absence of non-fitters in a previous cohort study [9] is compromised by our findings. The present study rather suggests also direct involvement of the corticospinal descending tract on the lower limb. However, the greater redundancy in descending pathways for the lower limb may be explained by the relative lower proportion of non-fitters (13%) when compared to the upper paretic limb (31%) in the current study.

The key challenge is to disentangle the underlying causes that define the 10 to 30% of the stroke patients that did not follow the proportional recovery rule, irrespective of initial lower or upper extremity motor deficit and irrespective of the involved neurological modality opposite of the hemispheric lesion [7]. Although stroke volume could intuitively be thought of as an important cause of not showing proportional recovery, Prabhakaran et al. (2008) showed that subcortical infarct volume was significantly related to change in FMA-UE scores in both fitters and non-fitters [3]. Other studies showed that there is no significant relation between lesion volume and proportional recovery of upper extremity motor function and language [8, 12]. It is suggested that the metabolic cascade (initially starting with energy failure due to hypo perfusion) that causes the intrinsic degeneration of distal axons, known as Wallerian degeneration, is fundamental to absence of spontaneous neurobiological recovery, as mechanisms that suppress spontaneous neurobiological recovery early after stroke are highly associated with disruption of the corticospinal tract [6, 13, 14]. However, one may also suggest that ‘abnormal network interactions’ suppress spontaneous neurobiological recovery, such as a deactivation to an anatomically related intact area or the changes in connectivity with this remote brain area (i.e., diaschisis) [15–19]. It could be hypothesized that, for example, when the connectivity in the brain network is not normalized, motor recovery is negatively influenced and patients do not show proportional recovery. In addition, potential factors that may limit neurobiological recovery are polymorphisms of the brain-derived neurotrophic factor (BDNF) gene [20–22], as well as blood-brain barrier dysfunction [23] that is associated with vasogenic edema [20]. We therefore advocate that the focus of future research should not only be on validating the proportional recovery rule and its intra-hemispheric generalizability for other affected modalities, but also on understanding underlying mechanisms of spontaneous neurobiological recovery. The ability of innovative pharmacological interventions to influence the proportion of non-fitters should be investigated as well. Examples are immunotherapy targeting the neurite growth-inhibitory protein Nogo-A [24, 25], therapies enhancing phasic GABA inhibition [26], and neural network modulating therapies [27]. Specifically, GABA (gamma-aminobutyric acid) is an inhibitory neurotransmitter that contributes to cortical functions, including motor control. Pharmacological agents may modulate phasic (synaptic) GABA signaling, which is suggested to enhance brain repair and plasticity related recovery after stroke [26]. In addition, pharmacological agents are also suggested to modulate non-invasive brain stimulation-induced network reorganization [27]. However, at this moment, we do not know which of these interventions are effective. Keeping in mind the suggested critical time window of recovery, these interventions should preferably be initiated within the first days poststroke [20]. Furthermore, research is needed to identify factors that hamper neurobiological recovery and which may lead to a relatively higher proportion of patients who do not follow the expected proportional recovery after stroke. For example, one may assume that high doses of early mobilization [28] enhances orthostatic variation in penumbral and oligemic brain areas early poststroke, and with that, may increase neurological damage early after stroke onset [29]. Therefore, neuro-imaging and neurophysiological determinants such as the quality of collateral blood flow defining early regional perfusion within the first 24 hours poststroke [30], the integrity of the affected corticospinal tract and its (a)symmetry index [31–34], stroke location (grey matter versus white matter), and dynamics in brain connectivity should be taken into account when investigating neurobiological markers of proportional recovery [33]. However, a recent systematic review showed that studies that use neurological biomarkers of brain impairment for prediction of motor recovery poststroke such as Diffusion Tensor Imaging, conventional structural MRI, and Transcranial Magnetic Stimulation need to improve their methodological quality in terms of cross-validation, considering the minimally clinical important difference of motor recovery, and recruitment of a large enough sample to provide sufficient statistical power [33]. In light of these recent findings, there is a need to underpin the added value of these neurological biomarkers next to behavioral markers in improving the predictive accuracy (i.e., true and false negatives) of fitters and non-fitters of the proportional recovery rule.

The first and main limitation of the present study is the exclusive use of clinical measures. Combining clinical with neuropsychological markers may improve prediction of neurobiological outcome [33], but further research needs to assess the cost-benefits of neuro-imaging measures in addition to clinical measures in predicting functional outcomes poststroke. Second, prediction of lower extremity recovery following the FMA is less precise than for the upper extremity [11]. Although the reliability of the FMA has been described as excellent [35], the measurement error for the lower extremity subscale is 6.4 points, resulting in a reliability change index of about 19% of the maximum score. In contrast, the measurement error for the upper extremity is 7.2 points, which is about 11% of the maximum score [11]. Consequently, this lack of precision makes the distribution of fitters and non-fitters along the estimated regression line in Fig 2 wider and more scattered when compared to the one for the paretic upper extremity [5]. In addition, due to the more scattered data points, using a different method to differentiate between fitters and non-fitters may have resulted in slightly differed groups. Third, we included only patients with a first-ever hemispheric ischemic stroke resulting in mild to moderate/severe neurological impairments at stroke onset. These patients may differ in the amount of pre-stroke comorbidities, as comorbidity is suggested to negatively influence outcome [36]. In addition, research by Ng and colleagues found that patients with multiple infarcts show spontaneous neurobiological recovery to a lesser extent than patients with a first-ever stroke [37], suggesting that quality of vascularization is an important issue for recovery. Contrary, a recent prospective cohort study did show that patients with previous or hemorrhagic strokes may also show proportional recovery of the upper extremity [38]. Fourth, although our patients received usual care according to prevailing guidelines, rehabilitation may have differed in intensity and type of therapy [39]. However, till so far, a number of studies failed to find evidence that type of therapy or intensity of practice interacts with spontaneous mechanisms of recovery [1, 6, 40]. Being more positive, high quality trials are needed to investigate if very early applied intensive therapies are able to affect the proportion of fitters and non-fitters of the proportional recovery rule.

Prospectively being able to identify patients who will fit or not fit the proportional recovery rule would influence both trial design and rehabilitation management dedicated to investigate the impact of services for the lower extremity [38, 41]. We already showed that stratifying patients based on expected spontaneous neurobiological recovery would have large consequences for the statistical power in stroke upper extremity trials as well as the choice for rehabilitation interventions [41]. For the lower limb, we showed that all patients who scored 14 points or more on the FMA-LE at baseline followed the proportional recovery rule. However, also 51 out of the 78 patients who scored less than 14 points showed proportional recovery for the lower limb. Consequently, this cut-off cannot simply be used to stratify patients. Due to the lack of statistical power for a multivariable regression analysis, we were not able to develop a clinical prognostic model within this subgroup of patients with an initial FMA-LE score below 14 points. Therefore, we recommend to further investigate this subgroup of patients with initially severe strokes and pool the current data with other (sub)cohorts with FMA-LE baseline scores below 14 points. Subsequently, factors could be identified that are able to distinguish between fitters and non-fitters. This will enable stratification of patients based on proportional recovery and with that, investigating the impact of stratification in trials on lower limb outcomes poststroke. Ideally, the 70% proportional recovery rule should be used in intervention trials by investigating interaction effects; aiming to increase the slope of the regression line (i.e., a higher percentage of proportional recovery) or to decrease the proportion of non-fitters. Above aims are in line with the recently published recommendations for improving stroke recovery and rehabilitation trials [42]. In parallel, our understanding of underlying mechanisms of spontaneous neurobiological recovery should be increased. Therefore, we need more translational research in which clinical, neuroimaging, molecular, and neurophysiological biomarkers of spontaneous neurobiological recovery are combined [42].
